# Evaluation of Genomic Selection for Seven Economic Traits in Yellow Drum (*Nibea albiflora*)

**DOI:** 10.1007/s10126-019-09925-7

**Published:** 2019-11-20

**Authors:** Guijia Liu, Linsong Dong, Linlin Gu, Zhaofang Han, Wenjing Zhang, Ming Fang, Zhiyong Wang

**Affiliations:** 1 Key Laboratory of Healthy Mariculture for the East China Sea, Ministry of Agriculture and Rural Affairs, Jimei University, Xiamen, China; 2 Laboratory for Marine Fisheries Science and Food Production Processes, Qingdao National Laboratory for Marine Science and Technology, Qingdao, China

**Keywords:** Genomic selection, *Nibea albiflora*, Predictive ability, GBLUP, Low-density SNPs

## Abstract

Yellow drum (*Nibea albiflora*) is an important maricultural fish in China, and genetic improvement is necessary for this species. This research evaluated the application of genomic selection methods to predict the genetic values of seven economic traits for yellow drum. Using genome-wide single-nucleotide polymorphisms (SNPs), we estimated the genetic parameters for seven traits, including body length (BL), swimming bladder index (SBI), swimming bladder weight (SBW), body thickness (BT), body height (BH), body length/body height ratio (LHR), and gonad weight index (GWI). The heritability estimates ranged from 0.309 to 0.843. We evaluated the prediction performance of various statistical methods, and no one method provided the highest predictive ability for all traits. We then evaluated and compared the use of genome-wide association study (GWAS)–informative SNPs and random SNPs for prediction and found that GWAS-informative SNPs obviously increased. It only needed 5 and 100 informative SNPs for LHR and BT to achieve almost the same predictive abilities as using genome-wide SNPs, and for BL, SBI, SBW, BH, and GWI, about 1000 to 3000 informative SNPs were needed to achieve whole-genome level predictive abilities. It can be concluded from the test results that breeders can use fewer SNPs to save the breeding costs of genomic selection for some traits.

**Electronic supplementary material:**

The online version of this article (10.1007/s10126-019-09925-7) contains supplementary material, which is available to authorized users.

## Introduction

Genomic selection (GS) uses genome-wide single-nucleotide polymorphisms (SNPs) to obtain breeding value estimates, which was first proposed by Meuwissen et al. (2001). The key feature of the GS is that it can provide more accurate estimates for kinship among individuals compared with the use of pedigrees (Muir 2007). In recent years, GS has been widely carried out in livestock breeding and has made great progress, especially for dairy cattle. It usually gives higher accuracy for breeding value estimates than the best linear unbiased prediction (BLUP), but the utilization of genomic selection in aquatic animals is very few (Hayes et al. 2009; Yue 2014; Dong et al. 2016a; Dou et al. 2016). The accurate selection for aquatic animals is also important due to the fact that one pair of aquatic animals usually produces a large number of fry and will have a big economic effect (Yue 2014). To date, genomic selection has already been applied to genetically improve the economic performance of many aquatic animals, such as Atlantic salmon (*Salmo salar*) (Tsai et al. 2015), sea bream (*Sparus aurata*) (Palaiokostas et al. 2016), Yesso scallops (*Patinopecten yessoensis*) (Dou et al. 2016), large yellow croaker (*Larimichthys crocea*) (Dong et al. 2016a), Pacific white shrimp (*Litopenaeus vannamei*) (Wang et al. 2017), the common carp (*Cyprinus carpio*) (Palaiokostas et al. 2018), Japanese flounder (*Paralichthys olivaceus*) (Liu et al. 2018), and so on.

Yellow drum (*Nibea albiflora*) is an economically important marine fish in China (Sun et al. 2018). Due to overfishing and environmental pollution, the number of wild populations has been dropped sharply (Han et al. 2008), and the Chinese government and breeders are now paying more attention to artificial breeding of yellow drum (Yang et al. 2013; Chen et al. 2017). Yellow drum is simply selected using phenotypic selection based on body size every year. Therefore, genetic progress has been quite limited. Furthermore, some economic traits cannot be measured without killing the fish, such as eviscerated weight and meat quality. Using genomic selection not only shortens the breeding cycle but also accelerates the genetic gain (Zenger et al. 2018). With the decrease in sequencing costs, it is easy to genotype genome-wide SNPs using whole-genome sequencing or genotyping-by-sequencing (GBS) (Rustagi et al. 2017). The genome of yellow drum is only approximately 565 Mb (Han et al. 2019), which is a great advantage for use with sequencing techniques to obtain a large number of genome-wide SNPs and favors the application of genomic selection to the yellow drum genetic breeding program. Genome-wide association study (GWAS) analysis is an efficient approach for screening trait-related markers or genes (Wang et al. 2019; Yu et al. 2019). In the future, we will further conduct GWAS analysis on these traits of yellow drum to further study the genetic mechanism of these traits.

In this research, we evaluate the application of genomic selection to the genetic improvement of seven yellow drum traits: body length (BL), swimming bladder index (SBI), swimming bladder weight (SBW), body thickness (BT), body height (BH), body length/body height ratio (LHR), and gonad weight index (GWI). The GBS technique was applied to genotype genome-wide SNPs in yellow drum, estimate genetic parameters for the above seven traits, and evaluate the predictive abilities of various genomic selection strategies.

## Materials and Methods

### Ethics Statement

All the yellow drums came from a fish breeding company Jinling Aquaculture Science and Technology Co. Ltd. in Ningde City, Fujian Province, P.R. China. This study was approved by the Animal Care and Use Committee of the Fisheries College of Jimei University.

### Sample Collection and Phenotype Measurement

In the spring of 2015, the parent fish were randomly selected from the Ningde Dongwuyang Bay. The parent fish were simultaneously injected with luteinizing hormone releasing hormone A3, and after 60 days of indoor breeding, the offspring were transferred to a floating cage for 1 year. Then, more than 6000 fish were randomly selected from the floating cage and transferred to the aquaculture farm. After 10 months of indoor rearing, a total of 393 yellow drums (195 males, 198 females) were collected as the experimental materials.

Severn traits were investigated, including BL, SBI, SBW, BT, BH, and GWI, which were measured in the Key Laboratory of Healthy Maricultural for the East China Sea. The weight of swimming bladder, gonads, and the body weight were measured using electronic scale with a precision of 0.01 g; the BL, BT, and BH were directly measured using vernier caliper with precision of 0.01 mm (for BT and BH) or 1 mm (for BL). Where SBI is the ratio of swimming bladder weight to body weight, LHR is the ratio of BL to BH, and GWI is the ratio of gonad weight to body weight.

### Genotyping and Quality Control

The fins of 393 fish were collected and the DNA was extracted with TIANamp Genomic DNA Kit (TIANGEN, Beijing, China); and DNA was sequenced with GBS technique implemented via Illumina HiSeq X Ten platform (Illumina, USA). After removing 22 samples with low coverage (< 3 × 10^6^), 371 samples (183 males and 188 females) were retained for SNP genotyping. The raw sequencing reads were filtered with FastQC software (Andrews 2010) and aligned to the reference genome (Han et al. 2019) using BWA v0.7.17software (Li and Durbin 2009); then, the SNPs were called with Platypusv0.8.1 (Rimmer et al. 2014), resulting in 3,868,328 SNPs. The SNPs were further filtered using plink v1.9 (Chang et al. 2015) with parameters “--vcf merge_pass.vcf --maf 0.01 --geno 0.1 --hwe 1e-5 --recode vcf -iid --biallelic-only --out snp --allow-extra-chr --threads 30”, resulting in 53,677 SNPs with the average missing rate 3.32%. The missing SNPs were imputed with software Beagle v4.1 (Browning and Browning 2016).

### Statistical Methods

All phenotypes are based on a linear model:

1 $$ \mathbf{y}=\mathbf{X}\boldsymbol{\upalpha } +\mathbf{B}\boldsymbol{\upbeta } +\mathbf{e}, $$

where **y** is the vector of the phenotypes for different traits; **X** is a design matrix for fixed effects; **α** is a vector of fixed effect (only the sex effect is included here); **B** is a design matrix for SNP effects (the elements in the SNPs genotypes are “0,” “1,” and “2” for genotypes “AA,” “Aa,” and “aa,” respectively); **β** is the vector for SNP effects; and **e** is the vector for residual effects. The residual effects are generally considered to be independent of each other and subject to the same distribution, **e** ∼ *N*(0, **Ι***σ*_*e*_^2^), where **I** is a vector of identity matrix and *σ*_*e*_^2^ is a residual variance. Genomic best linear unbiased prediction (GBLUP) (Vanraden 2008) directly predicted the genomic breeding values (GEBVs) of all individuals, and the mixed model equation can be expressed as

2 $$ \left[\begin{array}{l}{\mathbf{X}}^{\prime}\mathbf{X}\kern1.25em {\mathbf{X}}^{\prime}\mathbf{Z}\\ {}{\mathbf{Z}}^{\prime}\mathbf{X}\kern1.5em {\mathbf{Z}}^{\prime}\mathbf{Z}+{\mathbf{G}}^{-1}\lambda \end{array}\right]\left[\begin{array}{l}\hat{\boldsymbol{\upalpha}}\\ {}\hat{\mathbf{g}}\end{array}\right]=\left[\begin{array}{l}{\mathbf{X}}^{\prime}\mathbf{y}\\ {}{\mathbf{Z}}^{\prime}\mathbf{y}\end{array}\right], $$

where *λ* = *σ*_*e*_^2^/*σ*_*g*_^2^ = (1 − *h*^2^)/*h*^2^; *σ*_*g*_^2^ is the additive genetic variance of traits; *h*^2^ is the heritability of different traits, estimated from restrict-maximum likelihood (REML) method; and **Z** is the design matrix related to **g**. The **G** matrix can be obtained from all SNP genotypes:

3 $$ \mathbf{G}=\frac{\left(\mathbf{B}-2\mathbf{P}\right){\left(\mathbf{B}-2\mathbf{P}\right)}^{\prime }}{2\sum {p}_j\left(1-{p}_j\right)}, $$

where **P** is the vector of the allele frequency of each SNP and *p*_*j*_ is the frequency of allele “a” at the *j*th locus of each SNP.

We investigated 7 models for GS, BayesA (Meuwissen et al. 2001), BayesB (Meuwissen et al. 2001; Cheng et al. 2015), BayesCπ (Habier 2011), MMixp (Dong et al. 2017), ridge regression best linear unbiased prediction (RRBLUP) (Meuwissen et al. 2001), GBLUP (Vanraden 2008), and modified convolutional neural network (CNN) of Ma et al. (2018), where the CNN was modified to fit a large number of SNPs (unpublished method). In BayesB (Meuwissen et al. 2001; Cheng et al. 2015), the hyperparameter *π* (the probability of including an SNP in the model) is set as 0.001, 0.01, 0.1, and *v* (the degree of freedom of the inverse chi-square distribution) is set as 4.2 in this study. The scale parameter (*s*^2^) is derived by the expectation formula for the inverse chi-square distribution and *σ*_*g*_^2^. In the GBLUP and RRBLUP methods, the variance of all SNP effects is equal in the prior distribution (Meuwissen et al. 2001; Vanraden 2008). In BayesA, all SNPs have effects, and the variances of the SNPs follow inverse chi-square distribution (Meuwissen et al. 2001). MMixp also assumes different SNPs have different variance (large variance or small variance) (Dong et al. 2017). In BayesB and BayesCπ, only a small fraction of SNPs have non-zero effects (Meuwissen et al. 2001; Habier 2011; Cheng et al. 2015). The main difference between BayesB and BayesCπ is that the latter estimates the probability of the inclusion of a QTL into the model from Gibbs sampling but the former sets this parameter beforehand (Habier 2011; Cheng et al. 2015).

### Cross Validations

The cross-validation was used to test prediction accuracy. The 371 individuals were randomly divided into 2 groups (reference population and testing population) with the ratio about 5:1, resulting in a reference population containing ~ 334 fish and a testing population of ~ 37 fish. The populations were randomly sampled for 100 times, and for each sampling we evaluated the predictive ability for each strategy, where the predictive ability was calculated as the correlation coefficient between GEBVs and phenotypes.

## Results

### Genetic Parameter Estimates

The statistical results of phenotypic data of 7 traits are shown in Table 1. We tested the gender effect by comparing the phenotypes between the male and female group using *t* test for 7 traits. As shown in Table 1, gender has significant effect on the BL, GWI, SBI, BT, and BH, but not on the LHR and SBW. The phenotypes for the males and females of the sample sets are summarized in Table 1. There are significant differences for BL, SBI, BT, BH, and GWI between genders, suggesting that a gender effect must be considered in the estimation of breeding values. The variance components (*σ*_*g*_^2^ and *σ*_*e*_^2^) and heritability (*h*^2^) with 371 individuals were estimated using GVCBLUP software (Wang et al. 2014). The results are also presented in Table 1. The heritability of LHR was relatively lower (0.309 ± 0.140) compared with the other traits, and BL has the highest heritability (0.843 ± 0.150) among the 7 traits investigated (we will explain the overestimate of it in discussion). The heritability estimates of 6 of the 7 traits exceeded 0.4, suggesting these traits had the potentiality to be genetically improved.

**Table 1:** Statistical results for phenotypic data for seven traits

Trait	Male		Female		371 individuals		
	Number	Mean ± S.D.	Number	Mean ± S.D.	*σ*_*g*_^2^ ± S.E.	*σ*_*e*_^2^ ± S.E.	*h*^2^ ± S.E.
BL	183	211.80 ± 15.45 mm**	188	221.46 ± 16.08	237.123 ± 59.328	44.210 ± 39.475	0.843 ± 0.150
LHR	183	2.99 ± 0.21 (−)	188	2.99 ± 0.22	0.015 ± 0.007	0.032 ± 0.006	0.309 ± 0.140
SBI	183	0.72 ± 0.30%*	188	0.62 ± 0.42	556.844 ± 251.654	830.842 ± 200.971	0.401 ± 0.162
SBW	183	1.56 ± 0.68 g (−)	188	1.52 ± 0.84	0.301 ± 0.120	0.330 ± 0.092	0.477 ± 0.165
BT	183	35.81 ± 4.05 mm *	188	37.30 ± 3.98	11.923 ± 3.377	5.498 ± 2.379	0.684 ± 0.151
BH	183	71.14 ± 6.68 mm*	188	74.42 ± 5.95	29.656 ± 7.939	12.449 ± 5.540	0.704 ± 0.145
GWI	183	1.11 ± 0.43 %**	188	0.91 ± 0.25	0.104 ± 0.025	0.030 ± 0.017	0.773 ± 0.140

^*, **^Significant differences between sex at the 0.01 and 0.001 levels, respectively

### Performance of Different GS Methods

The predictive abilities for 7 traits are shown in Table 2. It was also noted that in BayesB (Meuwissen et al. 2001; Cheng et al. 2015), the hyperparameters have a great effect on prediction accuracy, and we chose the best one here. Using different algorithms, the predictive abilities ranged from 0.361 to 0.396 for BL, from 0.145 to 0.207 for LHR, from 0.203 to 0.242 for SBI, from 0.180 to 0.238 for SBW, from 0.283 to 0.317 for BT, from 0.350 to 0.400 for BH, and from 0.374 to 0.412 for GWI. The maximum difference and percentages of the differences across algorithms are also listed in Table 2. For some traits, such as BL and GWI, the difference among methods is small (< 10%), whereas for the other 5 traits, the differences were more than 10%, and as even as high as 24.4%. However, none of the methods consistently provided the highest predictive ability for all the traits. Among these traits, BL, BH, and GWI have higher prediction accuracy than the other traits.

**Table 2:** Effect of different methods on the predictive ability of 7 traits

Methods	Parameter	Predictive abilities (mean ± standard errors)						
		BL	LHR	SBI	SBW	BT	BH	GWI
BayesA		0.361 ± 0.011	0.157 ± 0.014	0.209 ± 0.014	0.184 ± 0.013	0.315 ± 0.017	0.355 ± 0.014	0.397 ± 0.013
BayesB	*π* = 0.001	0.240 ± 0.015	0.186 ± 0.014	0.177 ± 0.016	0.139 ± 0.017	0.259 ± 0.016	0.363 ± 0.012	0.349 ± 0.014
	*π* = 0.01	0.334 ± 0.015	0.186 ± 0.014	0.242 ± 0.018	0.191 ± 0.016	0.293 ± 0.015	0.393 ± 0.013	0.394 ± 0.012
	*π* = 0.1	0.362 ± 0.014	0.191 ± 0.014	0.230 ± 0.016	0.214 ± 0.015	0.298 ± 0.015	0.397 ± 0.013	0.400 ± 0.012
BayesCπ		0.396 ± 0.014	0.165 ± 0.014	0.203 ± 0.013	0.180 ± 0.014	0.317 ± 0.016	0.384 ± 0.015	0.391 ± 0.012
MMixp		0.378 ± 0.013	0.207 ± 0.014	0.215 ± 0.013	0.195 ± 0.012	0.296 ± 0.017	0.400 ± 0.012	0.386 ± 0.014
RRBLUP		0.380 ± 0.015	0.192 ± 0.014	0.224 ± 0.013	0.218 ± 0.013	0.298 ± 0.015	0.380 ± 0.012	0.389 ± 0.012
GBLUP		0.378 ± 0.015	0.174 ± 0.013	0.219 ± 0.014	0.238 ± 0.011	0.305 ± 0.017	0.365 ± 0.013	0.374 ± 0.014
CNN		0.392 ± 0.012	0.145 ± 0.015	0.207 ± 0.014	0.211 ± 0.016	0.283 ± 0.015	0.350 ± 0.014	0.412 ± 0.012
Maximum difference (percent of the difference)		0.035 (8.8%)	0.050 (24.2%)	0.039 (16.1%)	0.058 (24.4%)	0.034 (10.7%)	0.05 (12.5%)	0.038 (9.2%)

### Prediction Performance with Different Numbers of SNP Sets

To reduce the cost of selection, we evaluated the performance of genomic selection with an SNP set rather than using whole-genome SNPs. We built the SNP set in two ways. The first method was based on informative SNPs that were selected according to *p* values (from small to big) of GWAS that implemented via EMMAX software (Perry 2000; Meuwissen et al. 2001; Kang et al. 2010). The different number of SNPs were 5, 10, 15, 20, 30, 100, 200, 300, 400, 1000, 2000, 3000, 5000, 10,000, 20,000, 30,000, 40,000, and 53,677 (all). In contrast, the second method chose the same number of SNP set randomly. The GBLUP method (Vanraden 2008) was used to estimate the genetic values, and cross-validation was employed to test the predictive ability.

**Fig. 1:**
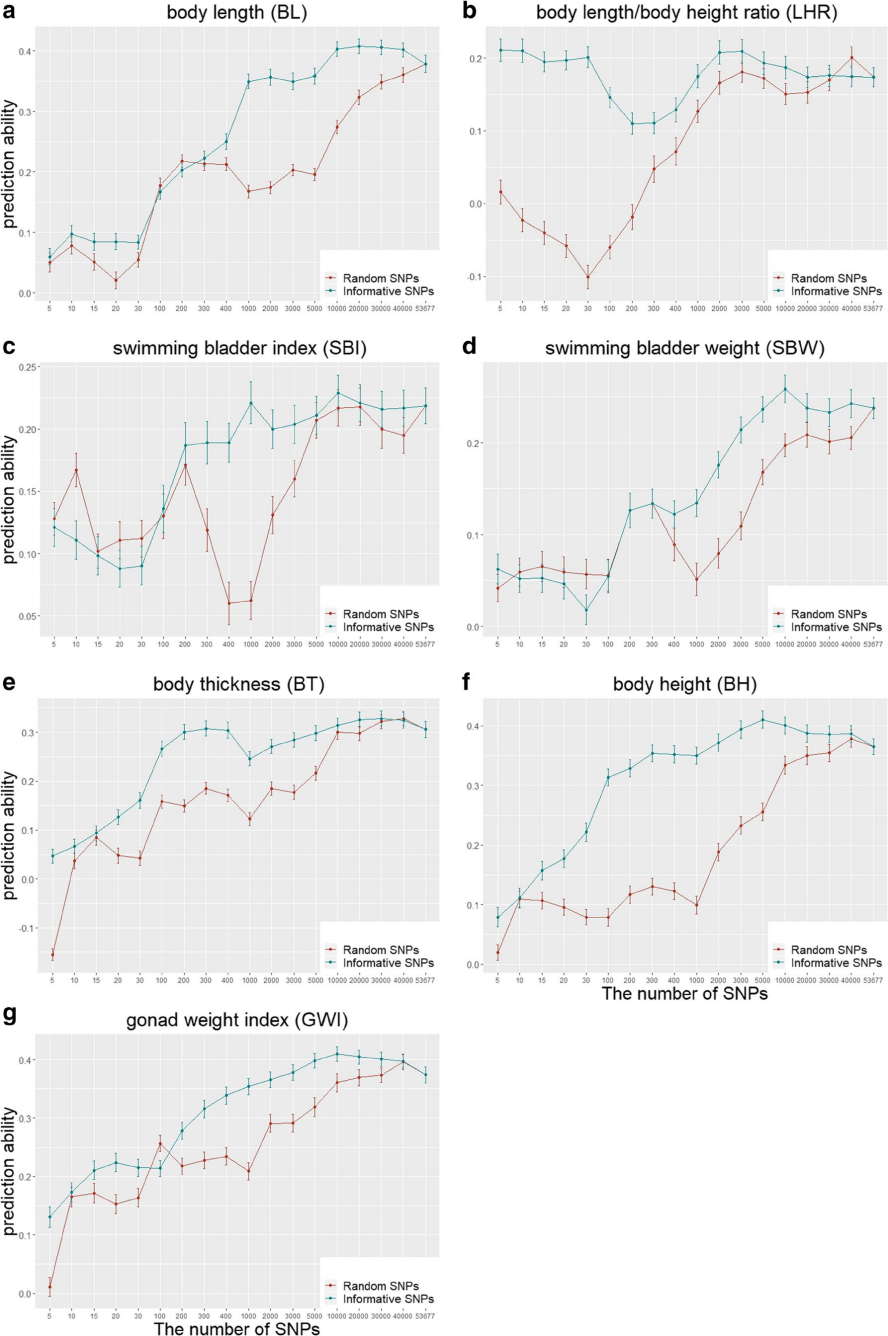
Predictive abilities under different numbers of GWAS informative SNPs and SNPs randomly selected by GBLUP

As shown in Fig. 1, generally, the predictive abilities were relatively higher with an informative SNP set than with a random SNP set, which suggests that informative SNPs are helpful in increasing predictive abilities; furthermore, for both strategies, the predictive abilities improved with an increase in the number of SNPs. Using 5 random SNPs, the predictive abilities were very close to zero or below zero for all traits, and compared with this, with the use of 5 informative SNPs, it produces more obvious predictive abilities for some of the traits, such as LHR, BT, and GWI. It is also shown in Fig. 1 that for BL, SBI, SBW, BH, and GWI, a set of 1000 to 3000 informative SNPs could bring almost the same predictive abilities as using the whole-genome SNPs; and for BT and LHR, it only needs approximately 100 and 5 informative SNP to achieve the same predictive abilities as using whole-genome SNPs. However, random SNP set requires much more SNPs (typically tens of thousands) to achieve similar predictive abilities to those achieved by using whole-genome SNPs. The results of LHR in Fig. 1b decreased from 30 to 2000 SNPs. The reason is that the sample size is small, and the pedigree of this population is complex, making the results a bit unstable, but which does not affect to get the general trend.

## Discussion

This research evaluated the performance of using genomic selection to predict breeding values for yellow drum, which provides a reference for future breeding programs. The study also successfully estimated the genetic parameters for 7 traits using genome-wide SNPs, which provides direct and accurate information to build kinship among individuals, and thus leads to accurate estimates for genetic parameters. Though heritability is usually estimated with pedigree information, by which the kinship among individuals can be built, it is very hard to get for yellow drum and they are also less accurate than using SNPs. The heritability estimates of BL, BH, and GWI, are 0.843, 0.704, and 0.773, respectively, and it seems that they are higher than expected value (0.4~0.6). Nevertheless, the 99% confidence intervals of the estimates are (0.457, 1), (0.330, 1), and (0.412, 1), respectively (obtained from the estimated heritability plus and minus two times of the standard error), showing that they include the expected value (0.4~0.6), suggesting the estimated heritability is within a statistically acceptable scope. In another hand, we only used a small number of fish (371) in this study and they were the offspring of several parents, maybe not representative for the whole population, and may lead to biased estimates for the heritability. The ideal population for the estimates of the heritability is a balanced population that consists as many families with similar number of offspring.

We also tested the prediction performance of different methods, including Bayesian methods, GBLUP (Vanraden 2008), and modified CNN method of Ma et al. (2018), but none always gave the highest predictive ability for all the traits. This suggests that when applying genomic selection for genetic breeding, we need to evaluate various methods and then choose the most suitable one for a specific trait (Dong et al. 2016b).

The size of the population is the most important factor affecting the predictive abilities. In this research, we only generated very small reference population (~ 334 fish), which led to low predictive abilities. The research inferred the size of the reference population using the method from Daetwyler et al. (2008). We fit the linear equation mentioned in Supplementary Table 2 of this paper and found that if we aim to achieve 0.8 of the prediction accuracy, then it needs 3773, 4206, 3621, 4606, 1640, 1702, and 2116 fish for BL, LHR, SBI, SBW, BT, BH, and GWI, respectively (see supplementary for detailed calculation). Another factor that affects the prediction ability is the genotyping methods. We used the GBS technique for SNP genotyping and generated ~ 50,000 SNPs, but it is still not dense enough to cover causal mutations. Compared with it, whole-genome sequencing is probably able to further increase the prediction abilities. The last factor that affects the prediction accuracy is the statistical methods; we have applied seven statistical methods for genomic selection in this study, and the results showed that the performance among methods (ranging from 8.8 to 24.4%) varied greatly. This suggests that statistical method has an ineligible effect on genomic selection. Hopefully, some sophisticated methods will be developed in the future that is able to further increase the predictive ability and suitable for more traits.

To conveniently evaluate the performance of SNP sets for genomic selection, we only investigated on the GBLUP method (Vanraden 2008). We compared the GWAS-informative SNPs to randomly selected SNPs and showed that GWAS-informative SNPs could bring predictive abilities as close as those using whole-genome SNPs, and the number of informative SNPs required ranged from 5 to 3000 depending on the traits. This conclusion is generally consistent with that of Abed et al. (2018) and Dassonneville et al. (2011), suggesting that one can use only a small number of informative SNPs rather than whole-genome SNPs for selection, which would dramatically reduce breeding costs (Habier et al. 2009; Dong et al. 2016a; Song et al. 2019).

## Electronic Supplementary Material

ESM 1 (DOCX 34 kb)

## Data Availability

Raw DNA sequencing reads were deposited in NCBI with the project accession of PRJNA533721.
