# Supplementary material for: Evaluation of Genomic Selection for Seven Economic Traits in Yellow Drum (Nibea albiflora)
Source: Mar Biotechnol (NY). 2019 Nov 20;21(6):806–12. doi: 10.1007/s10126-019-09925-7 (PMC6890617; doi:10.1007/s10126-019-09925-7)
Supplement: Supplementary file 1 — (DOCX 34 kb) [file 10126_2019_9925_MOESM1_ESM.docx]

# Supplementary Table

To solve the coefficient (b) of linear regression equation, this research set 15 levels, different numbers of training sets are randomly selected as follows: 26, 48, 70, 92, 114, 136, 158, 180, 202, 224, 246, 268, 290, 312, 334. The predictive abilities of different training sets were calculated with 15 levels, to solve the coefficient of linear equation. Different training sets were selected for the 7 traits, and the predictive ability was shown in Supplementary Table 1.

**Supplementary Table 1. Predictive ability to randomly select different numbers of training sets for 7 traits**

| Traits^a^ | Predictive ability of different number of training sets | | | | | | | | | | | | | | |
| --- | --- | --- | --- | --- | --- | --- | --- | --- | --- | --- | --- | --- | --- | --- | --- |
|  | 26 | 48 | 70 | 92 | 114 | 136 | 158 | 180 | 202 | 224 | 246 | 268 | 290 | 312 | 334 |
| BL | 0.097 | 0.190 | 0.184 | 0.236 | 0.247 | 0.284 | 0.260 | 0.295 | 0.320 | 0.351 | 0.327 | 0.344 | 0.349 | 0.326 | 0.358 |
| LHR | 0.073 | 0.079 | 0.092 | 0.113 | 0.155 | 0.141 | 0.141 | 0.182 | 0.099 | 0.144 | 0.168 | 0.176 | 0.218 | 0.203 | 0.167 |
| SBI | 0.085 | 0.116 | 0.136 | 0.154 | 0.171 | 0.175 | 0.185 | 0.198 | 0.175 | 0.172 | 0.192 | 0.192 | 0.244 | 0.227 | 0.247 |
| SBW | 0.093 | 0.090 | 0.066 | 0.077 | 0.111 | 0.088 | 0.084 | 0.187 | 0.195 | 0.193 | 0.204 | 0.158 | 0.220 | 0.188 | 0.202 |
| BT | 0.184 | 0.145 | 0.188 | 0.209 | 0.262 | 0.244 | 0.215 | 0.252 | 0.252 | 0.261 | 0.256 | 0.298 | 0.304 | 0.328 | 0.318 |
| BH | 0.164 | 0.200 | 0.218 | 0.271 | 0.306 | 0.281 | 0.301 | 0.343 | 0.347 | 0.327 | 0.334 | 0.336 | 0.382 | 0.346 | 0.351 |
| GWI | 0.164 | 0.169 | 0.257 | 0.279 | 0.271 | 0.286 | 0.315 | 0.329 | 0.345 | 0.375 | 0.418 | 0.380 | 0.381 | 0.392 | 0.396 |

a: The abbreviation means: body length (BL), body length/body height ratio (LHR), swimming bladder index (SBI), swimming bladder weight (SBW), body thickness (BT), body height (BH), gonad weight index (GWI); the unit is gram (g) for SBW; millimeter (mm) for BL, BT and BH; percentage (%) for SBI and GWI; LHR no unit; SBI = swimming bladder weight/body weight. GWI = gonad weight/body weight.

The ideal predictive accuracy was set at 80%, and the number of ideal training sets is shown in Supplementary Table 2.

**Supplementary Table 2. The ideal number of training sets when the prediction accuracy is 80%**

| Traits^a^ | Heritability | QTL^b^ | Equation | Number of ideal training sets |
| --- | --- | --- | --- | --- |
| BL | 0.843 | 1789.10 | $r_{(\hat{g},g)}=80\%=\sqrt{\frac{0.843N}{0.843N+1789.10}}$ | 3773 |
| LHR | 0.309 | 467.86 | $r_{(\hat{g},g)}=80\%=\sqrt{\frac{0.309N}{0.309N+467.86}}$ | 4206 |
| SBI | 0.401 | 522.73 | $r_{(\hat{g},g)}=80\%=\sqrt{\frac{0.401N}{0.401N+522.73}}$ | 3621 |
| SBW | 0.477 | 790.93 | $r_{(\hat{g},g)}=80\%=\sqrt{\frac{0.477N}{0.477N+790.93}}$ | 4606 |
| BT | 0.684 | 358.90 | $r_{(\hat{g},g)}=80\%=\sqrt{\frac{0.684N}{0.684N+358.90}}$ | 1640 |
| BH | 0.704 | 431.29 | $r_{(\hat{g},g)}=80\%=\sqrt{\frac{0.704N}{0.704N+431.29}}$ | 1702 |
| GWI | 0.773 | 588.61 | $r_{(\hat{g},g)}=80\%=\sqrt{\frac{0.773N}{0.773N+588.61}}$ | 2116 |

a: The abbreviation means: body length (BL), body length/body height ratio (LHR), swimming bladder index (SBI), swimming bladder weight (SBW), body thickness (BT), body height (BH), gonad weight index (GWI); the unit is gram (g) for SBW; millimeter (mm) for BL, BT and BH; percentage (%) for SBI and GWI; LHR no unit; SBI = swimming bladder weight/body weight. GWI = gonad weight/body weight.

b: Based on the results of equations (5) and Supplementary Table 1, the number of QTLs is inferred.
